# Longitudinal cognitive outcomes are strongly correlated with the Alzheimer’s Disease microbiome

**DOI:** 10.1002/alz.092640

**Published:** 2025-01-03

**Authors:** Abigail L Zeamer, Victoria Sanborn, Jonathan D Drake, John P Haran, Vanni Bucci

**Affiliations:** ^1^ University of Massachusetts Chan Medical School, Worcester, MA USA; ^2^ Alpert Medical School of Brown University, Providence, RI USA; ^3^ Brown University, Providence, RI USA

## Abstract

**Background:**

It has been shown that dysbiosis, or dysfunction of the gastrointestinal (gut) microbiome is associated with Alzheimer’s disease (AD). Here, we aimed to expand on beyond our previously reported findings of the gut microbiome associating with AD and explore if the gut microbiome is predictive of cognitive performance in individuals with AD. We sought to identity what cognitive domains are associated with the microbiome in our cohort of AD patients and healthy controls without dementia.

**Method:**

Older individuals residing in the general community of central Massachusetts were enrolled in our study. At each visit, fecal samples and clinical variables were collected in addition to cognitive testing using the ADAS‐Cog‐13 tool, such as delayed memory, word recall, recognition etc. Metagenomic profiling was performed on longitudinal fecal samples. Z‐scores for different cognitive domains, including memory, executive function and language were generated for the study population. Mixed‐effect random forest regression (MERFR) models were created to identify metagenomic features informative of cognitive performance across these different cognitive tests and domains.

**Result:**

Replicating our previous work, among AD diagnosed individuals, MERFR models predicted performance on ADAS‐Cog 13 from microbial abundance and pathways with a strong accuracy. The ADAS‐Cog 13 was not well predicted by the microbiome in the healthy controls. Additionally, in our new analysis across different cognitive domains, Z‐Scores were well predicted by MERFR models using microbial abundance and encoded pathways.

**Conclusion:**

Not only is the gut microbiome composition highly predictive of AD diagnosis, but there is also a strong correlation of the gut microbiome and cognitive functioning. This is true across the multiple domains of cognition including memory, executive function and language, however different bacterial species were significant in associating with each domain. This work highlights the complexity of the microbiome‐gut‐brain axis and how the microbiome community makeup might play a role in cognitive decline.

